# Assessment of human papillomavirus literacy among midwifery students: a cross-sectional study

**DOI:** 10.1590/1806-9282.20251205

**Published:** 2025-12-15

**Authors:** Emine Yeşilkaya, Maryam Nazhad Abbas, Taner Çelikel

**Affiliations:** 1 Süleyman Demirel University, Faculty of Health Sciences, Department of Midwifery – Isparta, Turkey.; 2 Faculty of Nursing, Department of Midwifery – Ankara, Turkey.; 3 Marmara University, School of Basic Medical Sciences, Department of Medical Biochemistry – Istanbul, Turkey.

**Keywords:** HPV, Literacy, Midwifery

## Abstract

**OBJECTIVE:**

Human papillomavirus, the primary etiological agent of cervical cancer, is considered to be the most common sexually transmitted infection worldwide. The aim of this study was to determine the human papillomavirus literacy levels of midwifery students.

**METHODS:**

This cross-sectional and descriptive study included midwifery students enrolled in a university in Türkiye during the 2024–2025 academic year. Out of 327 students enrolled in the midwifery program, 284 voluntarily participated in the study. Data were collected online using a Personal Information Form and the Human Papillomavirus Literacy Scale. A significance level of p<0.05 was used in the data analysis. Ethics committee and institutional approvals were obtained before the study.

**RESULTS:**

The mean age of the students was 21.08±2.46, and 27.8% of them were in the first year. A majority (82.7%) reported having information about human papillomavirus, primarily obtained from school (79.6%), social media/TV (48.6%), and health institutions (27.5%). Most students (97.5%) knew human papillomavirus is contagious, and 96.1% recommended human papillomavirus screening to others. The mean total Human Papillomavirus Literacy Scale score was 90.85±15.60, on a scale ranging from 24 to 120. The subdimension scores were as follows: 45.28±8.13 for knowledge, 21.75±4.56 for screening, and 23.81±4.04 for analysis.

**CONCLUSION:**

The study found that midwifery students have a high level of human papillomavirus literacy. The majority reported acquiring their knowledge through school and media, highlighting the positive impact of formal education and media on human papillomavirus awareness. The findings emphasize the importance of integrating human papillomavirus education into the curriculum. Additionally, community-based education programs can be effective in reaching broader populations, increasing awareness, and promoting preventive health behaviors.

## INTRODUCTION

Human papillomavirus (HPV) is the most common sexually transmitted infection worldwide and is the main causative agent of cervical cancer, causing approximately 600,000 new cases and 340,000 deaths each year^1^. The World Health Organization recommends HPV deoxyribonucleic acid (DNA) testing as the primary screening method for the general population^2^. HPV is usually transmitted by skin-to-skin or skin-to-mucosa contact and 80–90% of women encounter this virus at least once in their lifetime^3,4^.

HPV infections are mostly asymptomatic and cleared by the immune system, but up to 10% pose a risk of cervical oncogenic transformation^5,6^. HPV types are classified as high-risk (e.g., HPV-16, HPV-18) linked to cancers, especially cervical, and low-risk (e.g., HPV-6, HPV-11) causing benign conditions like genital warts^7^.

Up to 95% of cervical cancers result from persistent HPV infections. Sexual behaviors, including age at first intercourse and number of partners, affect HPV transmission risk. Despite available screening methods, access remains limited, particularly in low-resource settings^1,8^.

Vaccination, Pap smears, and HPV DNA tests are key in cervical cancer prevention. However, uncertainties about testing, vaccination, and treatment remain^8^. HPV prevention reduces morbidity, mortality, and social-economic burden. Midwives play a crucial role in assessment, counseling, and guidance^7,8^.

Limited research on HPV literacy levels of midwifery students is available in the literature. As future healthcare providers and young adults, midwifery students should possess high HPV knowledge and awareness. This study aimed to assess their HPV literacy levels.

## METHODS

### Study design and participants

This cross-sectional descriptive study was conducted in 2024–2025 with midwifery students at a state university in Türkiye's. The population included 327 students, and 284 who volunteered and completed the survey formed the sample. Using a 95% confidence level, 5% margin of error, and 50% response distribution, the minimum required sample size was calculated as 177. As the final sample exceeded this, it was deemed sufficient and representative.

### Data collection process

The data collection process was initiated by explaining the purpose of the research to the students at the end of the course. Participants were allowed to answer the questions, and assurance of confidentiality and anonymity was provided. The questionnaire form was filled in online via mobile devices, and each participant was restricted to answer only once. Participation was completely voluntary, and informed consent was obtained electronically.

### Data collection tools

Data were collected online using two tools: a Personal Information Form and the Human Papillomavirus Literacy Scale (HPV-LS).

### Personal Information Form

It was developed by the researchers in line with the literature and consists of 14 questions to determine the sociodemographic characteristics and health behaviors of the students^9,10^.

### Human Papillomavirus Literacy Scale

It was developed to measure the level of knowledge, understanding capacity and health-related decision-making skills related to HPV^11^. The scale consists of 24 items answered with a five-point Likert-type rating. The total score range is 24–120, with higher scores indicating higher HPV literacy. There are three subdimensions: Knowledge, Screening, and Analysis. The overall reliability coefficient of the scale was 0.97, and the Cronbach's α values for the subdimensions ranged between 0.88 and 0.95.

### Data analysis

Data were collected via Google Forms during the 2024–2025 academic year. Analyses used SPSS 27.0. Categorical data were shown as frequency and percentage; numerical data as mean±SD. Normality was tested with Kolmogorov-Smirnov test. One-way analysis of variance compared scale scores by demographics; Tukey HSD identified significant differences. Significance was set at p<0.05.

### Ethical considerations

Ethics committee approval was obtained from Süleyman Demirel University Health Sciences Ethics Committee on 13.02.2025 with decision number 91/3. Ethical principles were fully complied with in the study; written permission was obtained from the developers for scale use. The study also included the approval of the ethics committee of the relevant university.

### Findings

The mean age of the 284 students who participated in the study was 21.08±2.46 years, and 27.8% were first-year students. The income of 35.6% of the participants was covered by their families. Notably, 82.7% of the students stated that they had information about HPV, and 97.5% stated that they knew that HPV was contagious (Table 1).

**Table 1:** Sociodemographic characteristics of students (n=284).

Variables		Number	%
Age		mean±SD: 21.08±2.46 (min: 17; max: 38)	
Level of study			
	1st year	79	27.8
	2nd year	65	22.9
	3rd year	70	24.6
	4th year	70	24.6
Source of income			
	Family support	101	35.6
	Scholarship	80	28.2
	Student loan and supplementary scholarship	14	4.9
	Student loan and family support	67	23.6
	Student loan, scholarship, and family support	22	7.7
Do you know about HPV?			
	Yes	235	82.7
	No	49	17.3
Is HPV contagious?			
	Yes	277	97.5
	No	7	2.5

M: mean; SD: standard deviation; min: minimum; max: maximum; n: number of participants (n=284); HPV: human papillomavirus.

The mean total score of the Human Papillomavirus Literacy Scale was 90.85±15.60 (min. 24–max. 120). The mean score of the Knowledge Factor subdimension was 45.28±8.13, the mean score of the Screening Factor subdimension was 21.75±4.56, and the mean score of the Analysis Factor subdimension was 23.81±4.04 (Table 2).

**Table 2:** Students’ mean total scores of the Human Papillomavirus Literacy Scale and its subdimensions.

	Min	Max	Mean±SD	Cronbach's alpha
Knowledge factor	12	60	45.28±8.13	0.91
Screening factor	6	30	21.75±4.56	0.84
Analysis factor	6	30	23.81±4.04	0.87
Total score	24	120	90.85±15.60	0.95

M: mean; SD: standard deviation; min: minimum; max: maximum.

HPV-LS mean scores showed a significant difference according to grade level (p<0.05). Significant differences were found between the 1st grade and 3rd and 4th grades, and between the 2nd grade and 4th grade in the knowledge and screening subdimensions. Having information about HPV and recommending HPV screening significantly affected HPV-LS total and subdimension scores (p<0.05) (Table 3).

**Table 3:** The effect of sociodemographic characteristics of students on Human Papillomavirus Literacy Scale and its subdimensions.

Variables		Knowledge factor p/mean±SD		Screening factor p/mean±SD		Analysis factor p/mean±SD		Total scale p/mean±SD	
Year of study									
	1st year^a^	42.50	7.59	19.62	4.40	22.50	4.05	84.63	14.39
	2nd year^b^	44.20	6.60	21.23	3.52	23.89	3.59	89.32	12.42
	3rd year^c^	46.58	7.38	23.08	3.53	24.51	3.24	94.18	13.27
	4th year^d^	48.11	9.56	23.31	5.44	24.52	4.80	95.95	19.00
	f/p	7.33	**<0.001** (1st vs. 3rd, 4th) (2nd vs. 4th)	12.01	**<0.001** (1st vs. 3rd, 4th) (2nd vs. 4th)	4.33	**0.005** (1st vs. 3rd, 4th)	8.59	**<0.001** (1st vs. 3rd, 4th)
Source of income									
	Family support	43.99	6.87	21.11	4.01	22.90	3.95	88.00	13.24
	Student loan, scholarship and family support	44.00	13.62	20.90	7.43	22.72	7.26	87.63	27.67
	f/p	2.36	**0.05**	1.26	0.28	4.59	**<0.001**	2.68	**0.03**
Do you know about HPV?									
	Yes	46.81	7.37	22.62	4.16	24.64	3.38	94.08	13.85
	No	37.91	7.63	17.57	4.05	19.85	4.62	75.34	14.25
	t/p	7.63	**<0.001**	7.75	**<0.001**	8.40	**<0.001**	8.56	**<0.001**
Source of information about HPV (journal, book, and newspaper)									
	Yes	47.44	7.78	22.77	4.55	24.63	3.78	94.86	15.27
	No	44.72	8.14	21.49	4.53	23.60	4.09	89.82	1.55
	t/p	2.29	**0.011**	1.92	**0.028**	1.73	**0.042**	2.20	**0.01**
Source of information about HPV (university)									
	Yes	46.61	8.17	22.56	4.39	24.33	4.01	93.51	15.59
	No	43.51	7.76	20.67	4.57	23.13	3.99	87.31	14.96
	t/p	3.22	**<0.001**	3.53	**<0.001**	2.50	**0.006**	3.37	**<0.001**
Source of information about HPV (healthcare institution)									
	Yes	47.91	8.47	23.83	4.55	25.03	4.15	96.78	16.39
	No	44.28	7.79	20.96	4.32	23.35	3.91	88.60	14.72
	t/p	3.41	**<0.001**	4.91	**<0.001**	3.18	**<0.001**	4.04	**<0.001**
Would you recommend HPV screening to people around you?									
	Yes	45.69	7.86	21.91	4.46	24.03	3.80	91.64	14.95
	No	35.09	8.52	17.72	5.23	18.36	6.02	71.18	19.20
	t/p	4.37	**<0.001**	3.02	**0.001**	3.10	**0.005**	4.40	**<0.001**

M: mean; SD: standard deviation; Min: minimum; Max: maximum; HPV: human papillomavirus. p-values in bold are statistically significant (p<0.05).

a 1st Year (first-year midwifery student),

b 2nd Year (second-year midwifery student),

c 3rd Year (third-year midwifery student), and

d 4th Year (fourth-year midwifery student).

The HPV-LS and subdimension scores of the students who indicated school, magazines/books/newspapers, and health institutions as sources of information were significantly higher (p<0.05). While a significant relationship was found between the knowledge that HPV is contagious and HPV-LS total, knowledge, and analysis subdimensions, no significant difference was found in the screening subdimension. This shows that the effect of infectiousness knowledge on screening behavior is limited.

According to the source of income, a significant difference was observed in HPV-LS total, knowledge, and analysis subdimension scores only between students receiving "family support" and "family support+education loan" (p<0.05).

## DISCUSSION

Human papillomavirus is a common and usually asymptomatic sexually transmitted infection that affects men and women. Women have an 80–90% chance of being exposed to HPV during their lifetime, and more than 90% of cervical cancer cases are HPV-related. This cancer is most common in low- and middle-income countries where access to vaccination and screening services is limited. The WHO recommends vaccination between 9 and 14 years of age, screening after 30 years of age and early treatment for effective protection against HPV^5,6,7,8^.

Low HPV awareness hinders cancer prevention. Knowledge, screening behaviors, and health decisions are critical. Midwifery students are key as future health workers and a high-risk group. In our study, the total HPV literacy score of midwifery students was 90.85±15.60, and a high-level tendency was observed in all the information, analysis, and screening subdimensions. This shows that the students have a good level of knowledge in general.

A total of 82.7% of the participants reported having knowledge about HPV. This rate is consistent with the 90.3% rate reported in the study by Koçkaya et al.^12^. In the study by Soares Junior et al., 52.8% of all participants and 73% of female participants reported knowing HPV. While this percentage is similar for female participants, it is lower than the percentage in our study for the total number of participants. This is thought to be due to the fact that our study included only a group of female students studying in the field of health^13,14^.

The sources of information about HPV were determined as school, internet/TV, health institution, and written materials, respectively. Family and friends were the lowest source of information. Similarly, it has been reported in the literature that internet and social media ranked first, and family influence was low^10,15^. This may be explained by limited communication within families on sexual health issues. However, since misinformation can occur through media such as internet/TV, verified education programs are necessary. The analysis of HPV-LS scores showed that 3rd and 4th grade students scored significantly higher than 1st graders. This difference can be attributed to more theoretical and practical HPV training in advanced classes. Clinical practice and field experience also contribute to increased knowledge levels. Similarly, studies in Turkey reported significant differences in knowledge scores by class level.

The HPV-LS and subdimension scores of students who had knowledge about HPV and recommended HPV screening were significantly higher. Similarly, Huang et al. and Zhang et al. reported that the level of knowledge was positively associated with the behaviors of recommending and participating in screening^16,17^.

The higher HPV-LS scores of the students who preferred school, health institutions, and written sources as sources of information indicate the positive effect of information obtained from reliable sources on the literacy level.

It was observed that the knowledge that HPV is contagious affected the knowledge and analysis subdimensions, but not the screening subdimension. This suggests that awareness of infectiousness alone is not sufficient to direct to screening.

When the income status was analyzed, a significant difference was found in the total, knowledge, and analysis subdimensions of HPV-LS between students who received education only with family support and students who received both loan and family support. Similarly, in the studies of Kazancı et al. and Pruitt and Schootman, a positive correlation was found between parental income level and HPV knowledge score. As income increases, access to information and health awareness also increase^18,19^.

### Limitations of the research

Despite its valuable findings, this study is limited by its specific geographic region and time frame, which may affect the generalizability of the results to other settings or periods.

## CONCLUSION

The HPV literacy refers to individuals’ level of knowledge about HPV, their ability to understand this knowledge, and their ability to evaluate health-related decision-making processes. Therefore, it is believed that increasing public education and creating educational programmes aimed at raising HPV literacy in society will increase HPV literacy rates in society. Midwives, who are among the healthcare professionals who are in direct and constant communication with patients in the assessment of HPV, need to increase their knowledge of HPV in order to be able to assess individuals’ HPV status and provide the necessary health counseling.

## Data Availability

The datasets generated and/or analyzed during the current study are available from the corresponding author upon reasonable request.
